# A perspective on competitive freeride skiing and snowboarding

**DOI:** 10.3389/fphys.2025.1627889

**Published:** 2025-09-16

**Authors:** Eric Mulder, Hans-Christer Holmberg, Matej Supej

**Affiliations:** ^1^ Department of Health Sciences, Mid Sweden University, Östersund, Sweden; ^2^ Department of Physiology and Pharmacology, Biomedicum C5, Karolinska Institutet, Stockholm, Sweden; ^3^ School of Kinesiology, University of British Columbia, Vancouver, BC, Canada; ^4^ Division of Machine Elements, Department of Enginering Sciences and Mathematics, Luleå University of Technology, Luleå, Sweden; ^5^ Faculty of Sport, University of Ljubljana, Ljubljana, Slovenia

**Keywords:** avalanche safety, biomechanics, injury prevention, judging criteria, mental preparation, physiological demands, risk management, winter sport

## Abstract

Freeride skiing and snowboarding—collectively termed *competitive freeriding*—have evolved from niche extreme sports into formally recognized disciplines under the International Ski & Snowboard Federation (FIS). Unlike traditional alpine or freestyle events, competitive freeriding emphasizes creative line selection, technical execution, fluidity, style, and aerial maneuvers on natural, ungroomed mountain terrain. Athletes descend complex slopes based solely on visual inspection, without practice runs, facing unique physical and psychological challenges. This perspective article outlines the competition format and judging system, identifies key physiological and biomechanical demands, and reviews essential equipment and safety considerations. Despite growing popularity and institutional recognition, scientific research remains limited—primarily focused on avalanche risk and injury incidence—while other dimensions, such as psychological resilience, creative expression, and environmental connectedness, remain underexplored. Physiologically, competitive freeriders require high levels of eccentric and explosive strength, core stability, reactive control, and anaerobic endurance to navigate variable terrain and absorb impact during aerial maneuvers. Lower-extremity injuries—particularly anterior cruciate ligament (ACL) ruptures—are a major concern. Technological advances in drone-based filming, athlete monitoring, and protective equipment are reshaping freeride competition and broadcasting. As the sport moves toward potential Olympic inclusion, the central challenge lies in embracing innovation without compromising the core values of freedom, improvisation, and connection to the mountain environment.

## Introduction

Freeride skiing and snowboarding—collectively referred to here as *competitive freeriding*—involve descending natural, ungroomed mountain faces. Unlike alpine ski/snowboard racing, where athletes compete against the clock, freeriding is judged based on style, line choice, and technical execution (Freeride World Tour, 2025a). Emerging from the “extreme skiing” movement of the late 20th century, freeriding has rapidly evolved into a structured competitive discipline over the last 2 decades (Impiö and Parry, 2024; Tøstesen and Langseth, 2021). On 5 June 2024, during the 55th International Ski Congress in Reykjavik, Iceland, the International Ski & Snowboard Federation (FIS) formally recognized freeride as a distinct discipline (International Ski and Snowboard Federation [FIS], 2024) following FIS’s merger with the Freeride World Tour. This development marks freeriding’s transition toward mainstream recognition and further professionalization. While Olympic inclusion remains uncertain, FIS integration paves the way for expanded competition circuits, athlete development programs, and enhanced safety regulations (Impiö and Parry, 2024).

Despite its growing popularity (Vargyas, 2016), freeriding remains relatively underrepresented in scientific literature compared to other alpine disciplines. Most existing studies situate freeriding within the broader category of “extreme” or “high-risk” sports, emphasizing the inherent dangers of avalanche risk, steep terrain, and potential traumatic injury (Brymer, 2010; Hae et al., 2012; Niedermeier et al., 2020; Fruhauf et al., 2022). However, freeriding is not solely defined by risk; the sport is also characterized by a unique sense of freedom, creativity, and profound connection to natural mountain environments—attributes that have contributed to its growing popularity and to its classification by some authors as a “nature sport” (Impiö and Parry, 2024; Hornby et al., 2024).

Building on earlier exploratory work in risk-taking behaviour, psychological motivations, and the cultural dimensions of high-risk sports (Fruhauf et al., 2022; Hornby et al., 2024; Brymer and Schweitzer, 2013; Frühauf et al., 2017; Martinho et al., 2024), this article aims to provide a comprehensive perspective on competitive freeriding, covering its competition format, physical and physiological demands, judging criteria, safety measures and future challenges. By synthesizing existing knowledge and identifying gaps in the research, we highlight the need for further scientific exploration of competitive freeriding as an evolving sport as it transitions into the FIS framework.

## Competition format, judging system, and scoring criteria

Competitive freeriding is structured around a single-judged descent down a challenging, ungroomed mountain face (Freeride World Tour, 2025a). Unlike traditional alpine disciplines that rely on timed races or set courses, freeriding competitors select their route—often referred to as the “line”—making each run unique (Impiö and Parry, 2024). This fundamental characteristic ensures that style, technique, and terrain adaptation are key performance factors (Freeride World Tour, 2025a).

**FIGURE 1:**
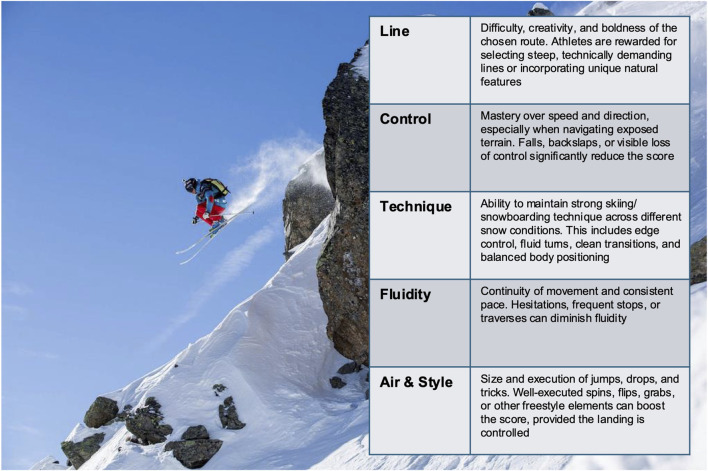
Illustration of the five judging criteria used in competitive freeride skiing and snowboarding—line choice, control, technique, fluidity, and execution of aerial maneuvers—as summarized in the accompanying table. Image provided by former FWT competitor Wille Lindberg; original photo by Freeride World Tour.

Freeride competitions occur in natural mountain environments characterized by unpredictable weather and snow conditions. Athletes dynamically navigate terrain features such as cliffs (Figure 1), wind lips, couloirs, and open faces (Impiö and Parry, 2024; Fruhauf et al., 2022). Unlike traditional alpine or freestyle events, freeriders rely solely on visual inspection—using drone footage, photographs, and personal observations—to memorize their intended route, without physical rehearsal on the course (Freeride World Tour, 2025a). The absence of practice runs significantly increases the mental demands, requiring athletes to visualize, anticipate, and adjust their line choice and riding style dynamically (Fruhauf et al., 2022). This lack of physical rehearsal not only heightens cognitive demands but also amplifies psychological pressure (Tøstesen and Langseth, 2021). Based on informal conversations and anecdotal accounts from athletes, freeriders often describe entering a heightened state of focus or “flow” during competition runs. While flow states have been studied in various sports (Jackson and Csikszentmihalyi, 1999), their role in freeride performance remains largely unexplored and could represent a valuable area for future research.

Competitors start individually from a designated point and must maintain fluidity and control throughout their descent. Although no strict time limit is imposed, prolonged hesitations or extensive traversing without decisive descent negatively impact scores. Performance is evaluated using an overall impression system based on five primary criteria (Figure 1). The judging panel typically consists of three to five experienced freeriders who apply a 0–100 scoring system to evaluate performance (Freeride World Tour, 2025a). Given the inherent subjectivity of freeride judging, consistency is reinforced through replay technology and multi-angle video review, particularly in close scoring situations (Ritter, 2023). Although the process mirrors other judged sports such as freestyle snowboarding and mogul skiing (Heiniger and Mercier, 2018), freeriding remains distinct due to its natural setting and open-ended performance structure.

Freestyle disciplines—including slopestyle and halfpipe—take place in controlled environments with engineered features and multiple scoring runs. These events prioritize amplitude, trick difficulty, execution, and landings, enabling more objective evaluation criteria (Flørenes et al., 2010). In contrast, freeriding unfolds on unpredictable mountain faces, where riders must select unique lines and execute a single, unrehearsed descent, intensifying psychological and technical demands (Freeride World Tour, 2025a; Fu et al., 2022). Both disciplines carry high injury risk, but while freestyle athletes primarily face repeated impacts from park features, freeriders contend with natural hazards such as rocks, cliffs, and variable snow conditions (Hornby et al., 2024; Flørenes et al., 2010).

Mogul skiing, another judged event, evaluates technical turning, aerial execution, and speed on a prepared slope (Flørenes et al., 2010). Freeride scoring, by contrast, bears closer resemblance to sports like surfing or skateboarding, where overall impression and risk-taking are integral components (Freeride World Tour, 2025a).

Ski mountaineering (SkiMo) provides another contrast, being a timed endurance discipline involving both ascents and descents on lightweight equipment (Müller et al., 2019; Bortolan et al., 2021). While it shares the off-piste setting and avalanche exposure with freeriding, it diverges markedly in its physiological demands, competition format, and gear (International Ski and Snowboard Federation [FIS], 2024). Freeriding’s non-linear structure accommodates a wide range of riding styles—from technical big-mountain skiing to trick-oriented descents—within a single judging framework (Freeride World Tour, 2025a).

### Physiological and physical demands

Freeride skiing and snowboarding places substantial physical demands on athletes due to the unpredictable terrain, technical complexity, and exposure to external forces. Unlike alpine disciplines with predefined courses, freeriders must navigate natural slopes while maintaining control and fluidity over jumps, drops, and steep sections. This requires a unique blend of technical skill, endurance, explosive power, balance, and reactivity. While the sport remains largely unexplored in scientific literature, meaningful comparisons can be drawn from related snow sports. Based on our observations, the most physically taxing elements often occur during rapid, reactive transitions—such as abrupt landings into variable snow or split-second redirections after cliff drops.

Freeriders rely heavily on eccentric, isometric, and explosive strength, particularly in the lower body and core. Eccentric strength and isometric control—key performance indicators in alpine racing—are likely essential for managing the substantial forces generated during landings, turns, and high-speed descents (Turnbull et al., 2009; Kröll et al., 2015; Franchi et al., 2019). In alpine skiing, ground reaction forces range from two to five times body weight (Gilgien et al., 2020; Supej et al., 2020), and similar or greater magnitudes are expected in freeriding, especially during cliff or jump landings. Stabilizing the body upon impact requires high eccentric hamstring strength (Färber et al., 2019) and core activation to avoid “back-seat” landings, which increase injury risk and result in score deductions. Core and upper-body control are also important for posture and impact absorption over rough terrain. The unpredictable snowpack demands rapid transitions between eccentric, isometric, and concentric muscle actions—underscoring the need for reactive and explosive strength. From a training perspective, this supports the need for integrated strength programs that simulate sudden directional changes and uncontrolled landings—scenarios not typically covered in alpine-specific regimens.

Competition runs in freeriding typically last 40 s to 3 min (personal observations), relying heavily on anaerobic energy. Studies in alpine skiing show a combination of anaerobic and aerobic demands, with glycolytic pathways supporting high-intensity efforts and aerobic endurance aiding recovery and altitude adaptation (Turnbull et al., 2009). In freeriding, aerobic fitness is especially relevant during ascents to start zones, such as the 3,223-m Bec des Rosses (Freeride World Tour, 2025a), while anaerobic capacity supports performance during the descent. As in mogul skiing, high lactate tolerance is likely critical to withstand repeated high-intensity efforts across a run (Turnbull et al., 2009).

Freeriding also presents a high risk of lower-limb injuries, particularly to the ACL, meniscus, and ankle joints due to frequent jumps, falls, and unpredictable impacts. In alpine skiing, ACL injuries commonly occur during suboptimal landings (Flørenes et al., 2010; Bere et al., 2014). Back-seat landings—where the skier lands with excessive hip flexion and leans too far backward—place increased stress on the knee, elevating the risk of ACL disruption in freeriders (Deady and Salonen, 2010).

Eccentric hamstring strength is essential for counteracting anterior tibial shear forces, thereby reducing ACL injury risk (Franchi et al., 2019). Coordinated activation of hamstrings and quadriceps enhances joint stability and impact absorption, particularly during landings on variable snow (Färber et al., 2019). Effective quadriceps–hamstring co-activation may be key in freeriding, where athletes must land smoothly despite inconsistent surface conditions.

Landing forces are closely linked to the athlete’s ability to manage impact. Axial forces correlate with knee joint loading, especially anterior tibial translation—a major contributor to ACL injury (Löfquist and Björklund, 2020; Yeow et al., 2011). Neutral landings with proper alignment reduce injury risk, while backward landings significantly increase ACL strain due to altered joint mechanics (Fu et al., 2022). Sex-based neuromuscular differences have also been implicated: women tend to exhibit greater knee valgus during landings, which may partly explain their higher ACL injury rates in alpine skiing (Cleather and Czasche, 2019).

### Equipment in competitive freeriding

**FIGURE 2:**
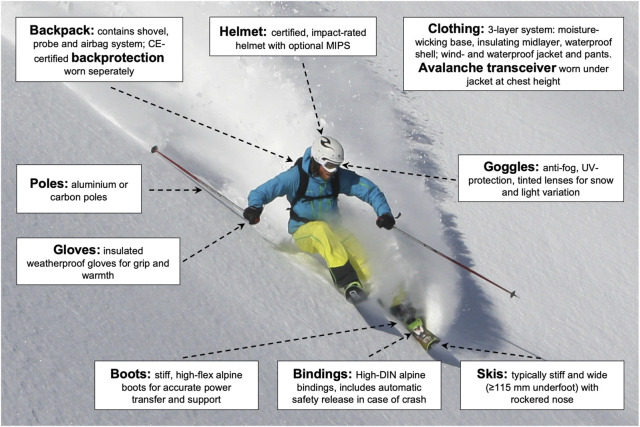
Major equipment features used in competitive freeride skiing. Athletes are equipped with specialized gear to manage high-impact landings, challenging snow conditions, and avalanche risk. A layered clothing system ensures thermal regulation and weather protection, while mandatory safety equipment includes an avalanche transceiver, airbag backpack, and helmet. Additional features such as integrated back protection, stiff skis and boots, and high-DIN bindings support control and impact absorption during steep descents and aerial maneuvers. Equipment must be worn and functional during the entire run and may be inspected before the start. The image is representative of freeride skiing but was not taken during an official competition. For illustration purposes, certain gear, such as the helmet-mounted camera, is not visible (photo by the first author, Eric Mulder).

Competitive freeride skiing and snowboarding necessitate equipment (Figure 2) designed to navigate steep, variable terrains while conforming to stringent safety standards (Freeride World Tour, 2025a). Although this equipment often resembles advanced backcountry gear, athletes participating in FIS-sanctioned events generally prioritize enhanced durability and stability (Müller et al., 2019). Freeride skis typically exceed 115 mm in width underfoot to provide flotation in powder and maintain control at high speeds (Freeride World Tour, 2023). These skis often incorporate advanced materials such as carbon fiber, Kevlar, or titanal to dampen vibrations and increase torsional stiffness (Müller et al., 2019). Developments in ski technology encompass innovations like rockered profiles, twin-tip designs, and 3D snowboard bases, which significantly enhance maneuverability in deep snow and broaden the range of achievable tricks. Research in vibration damping has also guided the development of “smart ski” prototypes with integrated damping mechanisms to improve stability on rough terrain (Schwanitz et al., 2018). Young athletes may initially use narrower skis (approximately 90 mm) before transitioning to wider variants, whereas snowboarders generally prefer stiff boards optimized for rapid descents (Müller et al., 2019; Eitzen et al., 2021).

Boots and bindings for both skiers and snowboarders emphasize support and retention. Freeride ski boots are typically constructed with rigid, alpine-style shells featuring high flex indices (e.g., 120–140) and are paired with bindings set to elevated ISO release values (Frühauf et al., 2017; Martinho et al., 2024; Ritter, 2023; Heiniger and Mercier, 2018; Flørenes et al., 2010; Fu et al., 2022) to prevent unintended release during high-impact maneuvers (Freeride World Tour, 2025a). This robust configuration is in stark contrast to ultralight mountaineering setups (Müller et al., 2019; Bortolan et al., 2021). Snowboarders generally select stiff boots and strap bindings to enhance control in challenging terrain (Collins et al., 2016). Recent advancements in boot technology, including heat-moldable liners and orthotic-based knee support, further improve control and reduce the risk of injury (Eitzen et al., 2021).

Safety considerations are paramount in freeride. Mandatory safety equipment includes a CE-certified back protector worn separately from the backpack, a helmet meeting advanced impact standards (e.g., EN 1077 Class B), avalanche transceiver (with fresh batteries), shovel, probe, avalanche airbag backpack in working order (with handle and leg strap fastened), and RECCO® reflector (Freeride World Tour, 2025b). Contemporary helmet designs frequently incorporate multi-directional impact protection (MIPS), carbon fiber shells, or optional full-face coverage. Avalanche airbags now utilize lightweight, reliable electric fan inflation systems to mitigate slide risks despite preventative measures at venues (Freeride World Tour, 2025a). Equipment checks are conducted before each run, and any missing or lost item results in disqualification. Additional gear such as harnesses or touring equipment may be required to access certain venues (Freeride World Tour, 2025b; Haines Borough, 2016).

### Injury incidence and risk factors

#### Injury incidence

Competitive freeriding is considered an extreme sport largely due to its substantial risk of traumatic injury (Tøstesen and Langseth, 2021; Brymer, 2010; Hornby et al., 2024). Given its unpredictable terrain and single-run format, the discipline is assumed to carry a high risk of serious injury. However, no large-scale epidemiological studies currently exist to quantify injury rates in competitive freeriding. Comparisons with other snow sports—such as freestyle skiing, where injury rates reach approximately 15.6 per 1,000 runs (Flørenes et al., 2010; Fu et al., 2022)—should therefore be approached with caution.

Anecdotal reports and media coverage suggest that season-ending injuries do occur among top-level freeriders, though these remain difficult to verify in the absence of centralized surveillance. This lack of systematic data underscores the need for improved reporting frameworks, especially as the sport becomes increasingly institutionalized. In our view, the integration of formal injury surveillance is not only timely but essential; adopting data-driven safety protocols—similar to those used in alpine disciplines—should be a priority to mitigate both acute and long-term injury risks.

#### Risk factors

The elevated injury risk in competitive freeriding arises from a confluence of interacting factors related to the environment, the activity itself, and the competitive context. These can be broadly categorized as follows:

1. Terrain features (hard surfaces and obstacles): A significant source of risk stems from the unforgiving nature of the terrain. Falls often result in impacts with hard and uneven surfaces, including cliff drops, exposed rocks, and other solid features. Furthermore, hidden obstacles beneath the snow surface such as rocks, tree stumps, and cliffs are widely recognized as inherent hazards that can cause unexpected collisions, dramatically increasing the severity of falls and injuries (Chalat, 1998).
2. Snow conditions (instability, avalanche risk and sluff): Freeriding takes place on slopes with variable and potentially unstable snowpacks. Instability significantly increases the risk of triggering avalanches, a major hazard that can lead to burial, trauma, and asphyxiation (Freeride World Tour, 2025a; Tøstesen and Langseth, 2021; Niedermeier et al., 2020; Fruhauf et al., 2022; Rong et al., 2025). Additionally, changing snow conditions across a single descent—ranging from soft to hard or icy surfaces due to altitude, wind, or sun exposure—can disrupt edge control and balance (Müller et al., 2019). Sluff (surface snow released by the skier’s own movements) may obscure terrain features and, in large volumes, create drag or secondary avalanche hazards (O'Bannon, 2012).
3. Slope characteristics and speed: The inherent nature of freeriding involves navigating steep slopes and often includes jumps, aerial maneuvers, and generally challenging terrain. These activities lead to high speeds, substantially increasing the kinetic energy involved in any impact. This elevated kinetic energy load exacerbates the potential for serious injury during falls or collisions. Biomechanical research in alpine skiing confirms that higher skier speeds and jump mechanics are closely associated with injury risk (Gilgien et al., 2019).
4. Competitive format: The judging-based format of freeriding events, which rewards difficulty, creativity, and risk, can incentivize athletes to attempt more challenging lines and maneuvers. This pursuit of higher scores may elevate their exposure to aforementioned hazards. Risk-taking in freeriding is not merely a by-product of the environment. It often functions as a deliberate strategy tied to recognition and competitive success (Tøstesen and Langseth, 2021). Athletes may therefore consciously push the limits of difficulty and danger to enhance their standing, navigating a complex interplay between performance ambition and personal safety. This behavior is not only personal, but also culturally embedded; within freeride communities, risk is frequently valorized as a marker of authenticity and skill, blurring moral boundaries between bravery, recklessness, and necessity (Tøstesen and Langseth, 2021; Brymer and Schweitzer, 2013). We believe these cultural dynamics warrant further qualitative study, especially as the sport grows more institutionalized under the FIS umbrella.
5. Inadequate or poorly adjusted equipment—such as boots, bindings, or avalanche gear—can reduce protection and elevate injury risk. High-impact landings and variable terrain place extreme demands on all gear components, making proper maintenance and fit essential. In alpine skiing, non-releasing bindings contribute to a large share of ACL injuries (Tarka et al., 2019), and over-tight settings—used to avoid premature release—are a known risk factor (Spörri et al., 2017). Although derived from alpine contexts, these findings are relevant to freeriding, where mechanical loads during jumps and variable snow conditions similarly challenge equipment function.
6. Freeriding demands a high level of physical conditioning, technical coordination, and terrain-reading skill. Inadequate strength, neuromuscular control, or insufficient recovery may reduce performance and impair decision-making in critical situations, thereby increasing injury risk. Studies in competitive alpine skiing have shown that prior injuries, fatigue, and deficits in physical preparedness contribute significantly to both the occurrence and severity of injuries—particularly in the lower extremities (Westin et al., 2012; Mancino et al., 2024). Additionally, emerging data from alpine and freestyle skiing also indicate sex-based differences in injury risk—particularly higher ACL rupture rates among female athletes, attributed to neuromuscular and biomechanical factors (Cleather and Czasche, 2019; Mancino et al., 2024). Whether similar patterns exist in freeriding remains unknown, but as female participation grows, this area warrants closer examination.

These risk factors, while categorized for clarity, often interact. For example, high speeds (factor 3) exacerbate the consequences of impacts with terrain features (factor 1) or hidden obstacles (factor 1). Similarly, unstable or rapidly changing snow conditions (factor 2) can be triggered by a rider’s own movement, particularly on steep slopes (factor 3). A comprehensive understanding of these interacting risks is crucial for developing effective injury prevention strategies. In our view, future studies should adopt a multidisciplinary approach that integrates biomechanics, snow science, and athlete psychology to better characterize injury mechanisms in this uniquely complex environment.

Injury risk is especially concerning among younger participants, where formal training structures may be lacking. Youth participation in competitive freeriding is steadily growing, as reflected by the increasing number of young athletes competing in events such as the Freeride Junior Tour (Fruhauf et al., 2022), which provides a global framework for entry into judged freeride competitions. However, athlete progression often remains informal and self-directed, with limited coaching oversight or formal avalanche training—factors that increase the risk of overexposure to dangerous terrain at a young age (Fruhauf et al., 2022).

Several promising models have, however, emerged at the regional level. In the United States, for example, the Jackson Hole Ski & Snowboard Club offers structured freeride programs that integrate technical training, safety education, and regular competition. In Sweden, a recently proposed public upper secondary school program dedicated to freeriding in Åre (Johansson, 2025) marks a move toward institutionalizing access to training. Building on these developments, we suggest that future research and policy focus on scalable models for youth engagement—combining skill progression frameworks, avalanche awareness, and psychological preparation. Furthermore, freeriding remains a resource-intensive sport, requiring significant investment in equipment, travel, and coaching—factors that may restrict broader youth participation (Fruhauf et al., 2022; Hudson et al., 2010). Improving access will require broader efforts to support regional clubs, reduce equipment-related barriers, and incorporate freeride modules into national ski education systems.

### Video footage, data analysis and broadcasting

Although freeriding remains grounded in the unpredictability of natural terrain, recent technological innovations have begun to reshape equipment design, safety protocols, and performance analytics (Freeride World Tour, 2025a; Eitzen et al., 2021). Helmet-mounted cameras are now mandatory (Freeride World Tour, 2025b), with GoPro (San Mateo, CA, United States) as the official partner, fully integrated into rider gear (GoPro, 2025). High-resolution drone footage and live streaming have transformed how runs are documented and judged (Freeride World Tour, 2025a; Hornby et al., 2024). Organisers can provide multi-angle replays to support more precise scoring, while athletes and coaches gain valuable visual feedback for evaluating line choice and technique. Enhanced broadcasting capabilities have elevated global viewership and sponsorship, supporting both sport visibility and athlete infrastructure (Müller et al., 2019).

In the future, competitions may integrate real-time athlete metrics—such as heart rate or impact forces—into live streams, deepening audience engagement and reinforcing freeriding’s status as a data-informed, rapidly evolving discipline. GPS trackers and wearable inertial measurement units (IMUs) are also under consideration, potentially enabling the collection of speed, jump height, and acceleration data during runs (Eitzen et al., 2021). Differential global navigation satellite system technology (dGNSS) systems have previously demonstrated high precision in alpine skiing settings for capturing velocity, trajectory, and external loads (Gilgien et al., 2013), offering a promising model. However, the application of such tools in freeride environments presents unique challenges, including reduced GPS accuracy due to signal obstruction from cliffs or dense topography, and the difficulty of securely attaching sensors without compromising mobility (Camomilla et al., 2018). Despite these hurdles, such technologies could eventually provide valuable insights for both performance optimization and injury risk management in a high-consequence sport. In our view, the integration of real-time athlete data into broadcasts not only enhances viewer experience but also holds untapped potential for injury prevention and skill progression analysis.

Together, these technological developments reflect freeriding’s evolving balance between tradition and innovation. Even as equipment, analytics, and safety systems advance, the core appeal—navigating untracked slopes and daunting cliffs—remains unchanged. Emerging tools such as AI-assisted judging software or computer vision-based edge detection—already explored in other action and judged sports (Cossich et al., 2023)—may also assist in validating rider position and trick execution in real-time, helping to supplement subjective assessments. As freeride becomes more embedded within the FIS competition framework, we believe that continued research and investment in cutting-edge tools will shape the next-generation of the sport.

### Future perspectives

Freeride’s formal recognition by FIS in 2024 (International Ski and Snowboard Federation [FIS], 2024) has sparked discussion about its potential inclusion in the Winter Olympic Games, with trial or demonstration events likely to assess feasibility. We believe Olympic inclusion would mark a major milestone, but it would also require standardized judging frameworks and reproducible venues—objectives that must be balanced with freeriding’s foundational ethos of creativity and natural terrain (Impiö and Parry, 2024).

Beyond the Olympic debate, FIS involvement is expected to expand the competitive landscape; additional national and continental circuits may emerge, potentially supported by standardized junior development frameworks. These systems could reinforce risk awareness and technical progression, aligning freeriding more closely with institutional models seen in alpine racing (Impiö and Parry, 2024).

As the sport evolves, injury surveillance and wearable sensors may play a greater role in monitoring acting ground reaction forces and head impacts, enhancing medical response and athlete safety (Müller et al., 2019; Eitzen et al., 2021). Advances in 3D mapping and virtual reality may soon allow athletes to rehearse line selection digitally, supporting both performance optimization and risk management (Cossich et al., 2023). We believe that the rapid evolution of sport technology in freeriding underscores the need for proactive, athlete-informed research to guide ethical, performance-driven integration. Improvements in GPS tracking, motion analysis, and biomechanical data collection may also contribute to more objective judging—providing quantifiable insights into speed, aerial execution, and impact forces. For instance, IMUs could assist in validating jump rotations, while force sensors embedded in boots or skis might quantify impact absorption (Eitzen et al., 2021). Nonetheless, successful integration depends on overcoming several barriers, including sensor calibration, data standardization, and environmental interference (Camomilla et al., 2018). From our perspective, developing robust, field-ready sensor systems tailored to the demands of backcountry competition presents a key opportunity for innovation in freeride sport science. However, as freeriding becomes increasingly integrated into the FIS framework, maintaining a balance between competitive structure and the sport’s core values of creativity, improvisation, and mountain exploration remains essential. With this new institutional legitimacy, however, comes the risk of marginalizing local subcultures that have historically shaped the sport’s identity. Preserving these diverse traditions—including community-led competitions, backcountry exploration, and the celebration of individual style—will be critical to safeguarding the sport’s cultural richness.

## Conclusion

Competitive freeride skiing and snowboarding exemplify the boundary between high-stakes risk, technical proficiency, and creative expression—blending elements of freestyle, big-mountain exploration, and individual style (International Ski and Snowboard Federation [FIS], 2024; Brymer, 2010; Hornby et al., 2024). With formal recognition by FIS, the sport is entering a new phase of institutional integration, with expanded competition circuits and potential Olympic inclusion on the horizon. These developments, along with advancements in equipment, judging systems, and injury monitoring, are already beginning to reshape freeriding’s competitive landscape. The challenge ahead lies in adopting innovation without compromising the sport’s foundational values: freedom, spontaneity, and deep connection to the natural environment. As freeriding evolves, preserving this balance will be key to its sustained growth and authenticity.

## Data Availability

The original contributions presented in the study are included in the article/supplementary material, further inquiries can be directed to the corresponding author.
